# What are the benefits of parental care? The importance of parental effects on developmental rate

**DOI:** 10.1002/ece3.1083

**Published:** 2014-05-12

**Authors:** Hope Klug, Michael B Bonsall

**Affiliations:** 1 Department of Biological & Environmental Sciences, University of Tennessee at Chattanooga215 Holt Hall, Dept 2653 615 McCallie Aven, Chattanooga, 37403, Tennessee; 2 Mathematical Ecology Research Group, Department of Zoology, University of OxfordOxford, OX1 3PS, U.K; 3 St Peter's CollegeOxford, OX1 2DL, U.K

**Keywords:** Hatching plasticity, life history, offspring performance, parental care, parental effects, parental investment, safe-harbor hypothesis

## Abstract

The evolution of parental care is beneficial if it facilitates offspring performance traits that are ultimately tied to offspring fitness. While this may seem self-evident, the benefits of parental care have received relatively little theoretical exploration. Here, we develop a theoretical model that elucidates how parental care can affect offspring performance and which aspects of offspring performance (e.g., survival, development) are likely to be influenced by care. We begin by summarizing four general types of parental care benefits. Care can be beneficial if parents (1) increase offspring survival during the stage in which parents and offspring are associated, (2) improve offspring quality in a way that leads to increased offspring survival and/or reproduction in the future when parents are no longer associated with offspring, and/or (3) directly increase offspring reproductive success when parents and offspring remain associated into adulthood. We additionally suggest that parental control over offspring developmental rate might represent a substantial, yet underappreciated, benefit of care. We hypothesize that parents adjust the amount of time offspring spend in life-history stages in response to expected offspring mortality, which in turn might increase overall offspring survival, and ultimately, fitness of parents and offspring. Using a theoretical evolutionary framework, we show that parental control over offspring developmental rate can represent a significant, or even the sole, benefit of care. Considering this benefit influences our general understanding of the evolution of care, as parental control over offspring developmental rate can increase the range of life-history conditions (e.g., egg and juvenile mortalities) under which care can evolve.

## Introduction

Patterns of parental care are hugely diverse (Ridley 1978; Baylis 1981; Tallamy 1984; Clutton-Brock 1991: Rosenblatt 2003; Balshine 2012; Royle et al. 2012; Trumbo 2012), and parental care is thought to have emerged independently multiple times (Rosenblatt and Snowdon 1996; Mank et al. 2005). A large amount of work has focused on identifying the conditions under which some form of parental care from an ancestral state of no care will evolve (e.g., Sargent et al. 1987; Clutton-Brock 1991; Winemiller and Rose 1992; Webb et al. 2002; Mank et al. 2005; Kokko and Jennions 2008; Klug and Bonsall 2010; Klug et al. 2012, 2013). Most generally, the evolution of parental care is expected to be favored when the fitness benefits to the caring parent(s) outweigh the costs associated with care (e.g., reduced parental survival or future reproduction).

Parental care is beneficial to parents if it increases offspring survival, growth and/or quality (i.e., offspring performance), and ultimately offspring lifetime reproductive success (Clutton-Brock 1991; Rauter and Moore 2002; Alonso-Alvarez and Velando 2012; Klug et al. 2012; Royle et al. 2012). There are several general and nonmutually exclusive ways in which parental care can be beneficial, which we review in Table 1. In summarizing and categorizing the benefits of care, we focus on (1) the specific life-history stage(s) in which care is beneficial and (2) the specific way in which care benefits offspring, as both of these factors have been shown to influence the conditions under which care can originate (Alonso-Alvarez and Velando 2012; Klug et al. 2012).

**Table 1:** Benefits of parental care. We summarize four general types of parental care benefits, list mechanisms that might give rise to such benefits, and provide empirical examples from recent work on benefits of care. In categorizing benefits of care, we focus on (1) the specific life-history stage(s) in which care is beneficial and (2) the specific way in which care benefits offspring, as these factors have been shown to influence the conditions under which care can originate. Additionally, we primarily use empirical examples from studies that have documented benefits of care since the publication of Clutton-Brock's book (1991) on the evolution of parental care. It is important to note that this is not an exhaustive list of possible benefits of parental care, and many forms of care are likely to be associated with more than one type of benefit

General Benefit
**(1) Parents ↑ offspring survival during the stage in which parent and offspring are associated**
Mechanisms & Examples
(a) Protection from predators (e.g., defensive behavior, increased vigilance, offspring carrying, alarm calls, mate guarding, dilution effects)
• Males defend eggs in the pine engraver bark beetle (*Ips pini*, Reid and Roitberg 1994), Puerto Rican cave-dwelling frog (*Eleutherodactylus cooki*, Burrowes 2000), flagfish (*Jordanella floridae*, Klug et al. 2005; Hale 2008), and harvestman (*Iporangaia pustulosa*, Requena et al. 2009).
• Maternal defense of eggs, nymphs or juveniles occurs in the European earwig (Kolliker 2007), spider *Coelotes terrestris* (Gundermann et al. 1997), the amphipods *Leptocheirus pinguis*, *Casco bigelowi*, and *Dyopedos monacanthus* (Thiel 1999), treehoppers (*Publilia concave*, Zink 2003), harvestman (*Acutisoma proximum*, Buzatto et al. 2007), burrower bugs (*Adomerus triguttulus*, Nakahira and Kudo 2008), and mountain goats (*Oreamnos americanus*; Hamel and Cote 2009).
• Both parents protect young against predation in tree swallows (*Tachycineta bicolor*, Winkler 1992), spotted tilapia (*Tilapia mariae*, Annett et al. 1999), Siberian jays (*Perisoreus infaustus*, Griesser 2003), and black rock skinks (*Egernia saxatilis*, O'Connor and Shine 2004; Langkilde et al. 2007).
• Male gladiator frogs protect eggs from being destroyed by other males, thereby improving egg survival (*Hyla faber*, Martins et al. 1998).
• Adoption of foreign young in Convict cichlid reduces predation on parents' own offspring through dilution effects under some conditions (*Cichlasoma nigrofasciatum*, Fraser and Keenleyside 1995).
• Alarm calling in the yellow-bellied marmot (*Marmota flaviventris*) increases offspring survival (Blumstein et al. 1997).
• Foot drumming by kangaroo rat mothers reduces stalking by snake predators (*Dipodomys spectabilis*, Randall and Matocq 1997).
• Maternal vigilance in feral horses protects mares from infanticide (*Equus caballus*, Cameron et al. 2003).
• Female beetles add a coating to eggs after laying which reduces predation on eggs (*Cryptocephalus hypochaeridis*, Ang et al. 2008).
• Females of the amphipod *Apherusa jurinei* retrieve embryos that are removed from the brood pouch (Wheeler 2008).
• Mate guarding by males is associated with increased female incubation in two songbirds, which presumably increases offspring survival (*Junco hyemalis caniceps* & *Cardellina rubrifrons;* Fedy and Martin 2009).
(b) Provisioning (e.g., lactation, preparation of food, feeding of captured prey)
• Female European earwigs regurgitate food to their nymphs (*Forficula auricularia*, Kolliker 2007; Staerkle and Kolliker 2008)
• Great tit parents prepare food for offspring, which likely facilitates ingestion and digestion of prey (*Parus major*, Barba et al. 1996).
• Mothers of the spider *Coelotes terrestris* increase offspring survival by supplying their young with food (Gundermann et al. 1997).
• Burying beetle parents defend carcasses and regurgitate food to larvae, thereby increasing larval survival (*Nicrophorus vespilloides*, Eggert et al. 1998).
• Male and female Australian magpie-larks feed nestlings (*Grallina cyanoleuca*, Hall 1999).
• Ant tending by treehopper mothers increases offspring survival (*Publilia modesta*, Billick et al. 2001).
(c) Reduced risk of egg dehydration or offspring desiccation
• Paternal care in the Puerto Rican frog *Eleutherodactylus coqui* prevents mortality due to egg desiccation (Townsend et al. 1984).
• Males of the desert beetle *Parastizopus armaticeps* maintain high moisture levels in burrows, which is essential for offspring survival (Rasa 1998).
• Seedling association with maternal tissue increases survival in two alpine plants, *Frasera speciosa* and *Cirsium scopulorum* (Wied and Galen 1998).
• Egg brooding in Children's pythons reduces embryonic water loss and promotes egg viability (Lourdais et al. 2007; Stahlschmidt et al. 2008)
(d) Offspring waste removal (e.g., feces eating)
• Parental tree swallows (*Tachycineta bicolor*), red-winged blackbirds (*Agelaius phoeniceus*), and American robins (*Turdus migratorius*) eat or remove fecal sacks of offspring (Hurd et al. 1991).
(e) Increased egg oxygenation (e.g., fanning, brood pumping)
• Male sand gobies fan their eggs until hatching and adjust the level of fanning in response to dissolved oxygen and nest structure (*Pomatoschistus minutus*, Lissåker and Kvarnemo 2006; Järvi-Laturi et al. 2008).
• Waterbug males exhibit brood pumping that oxygenates eggs and increases hatching success (*Abedus breviceps*, Munguia-Steyer et al. 2008).
(f) Offspring physiochemical adjustment
• Female bromeliad crabs use shells to adjust Ca^2+^ and pH, which is necessary for offspring development and survival (*Metopaulias depressus*, Diesel 1997).
(g) Increased offspring immune function
• The presence of the father at great tit nests increases nestling immune response (*Parus major*, Tinne et al. 2005).
(h) Protection from parasites, parasitoids, and disease
• Mothers of the spider *Coelotes terrestris* protect their young against parasites (Gundermann et al. 1997).
• Female marbled salamanders decrease fungal infection at the nest, which increases hatching success (*Ambystoma opacum*, Croshaw and Scott 2005).
• Male egg carrying in the golden egg bug *Phyllomorpha laciniata* protects eggs against parasatoids (Gomendio et al. 2008).
• Peacock blenny fathers produce secretions that protect eggs from bacterial infection and increases egg survival (*Salaria pavo*, Pizzolon et al. 2010)
(i) Reduction of offspring energetic expenditure (e.g., carrying, thermoregulation)
• Nest maintenance by male chinstrap penguins improves thermal nest characteristics (*Pygoscelis antarctica*, Fargallo et al. 2001).
• Male care in fat-tailed dwarf lemurs is thought to have thermoregulatory benefits to offspring (*Cheirogaleus medius*, Fietz and Dausmann 2003).
• Echelon position in dolphins (i.e. calf in close proximity to the mother's mid-lateral flank) reduces calf swimming effort, which allows mother and offspring to remain in close proximity; close proximity to the mother is thought to be vital for infant survival (*Tursiops truncates*; Noren et al. 2008).
(j) Behavioral support of offspring during intra-specific interactions
• Juvenile black rock skinks receive foraging and thermoregulatory benefits that are related to their parents' status (O'Connor and Shine 2004).
(k) Teaching or facilitation of learning
• Pied babblers parents condition offspring to associate purr calls with food; parents then use this association to cause fledglings to move toward food sources (*Turdoides bicolor*; Raihani and Ridley 2008).
**(2) Parents improve some aspect of offspring quality, which leads to an ↑ in offspring survival and/or reproductive success in the future (i.e., when parents and offspring are no longer associated)**
Mechanisms & Examples
(a) *Provisioning* (e.g., lactation, preparation of food, feeding of captured prey)
• Burying beetles (*Nicrophorus vespilloides*) defend carcasses and regurgitate food to their larvae; this increases larval mass (Eggert et al. 1998).
• Female red squirrels store food prior to mating and provide these stores to offspring at independence (*Tamiasciurus hudsonicus*, Boutin et al. 2000).
• Matriphagy in the foliage spider *Chiracanthium japonicum* increases offspring weight gain and predispersal survival (Toyama 2001)
• Length of the rearing period in kittiwakes is positively correlated with survival and future reproductive performance (*Rissa tridactyla*, Cam et al. 2003)
• Females of the spider *Stegodyphus lineatus* provide young with regurgitated food and water and eventually their body; maternal provisioning affects offspring mass at dispersal, which is likely to affect future fitness (Salomon et al. 2005).
• Females of the cichlid *Tropheus moorii* feed their young in their mouth; this maternal feeding increases offspring size, weight, and swimming speeds, which is expected to affect subsequent survival (Schurch and Taborsky 2005).
• Paternal presence at the nest increases offspring body mass and likelihood of breeding the following year in nestling great tits (Tinne et al. 2005).
• Parental pied babblers use purr calls to direct fledglings toward food sources; this provisioning is expected to lead to heavier offspring that are more likely to reproduce as adults (*Turdoides bicolor*; Radford and Ridley 2006).
(b) Reduced risk of egg dehydration or offspring desiccation
Ball python egg brooding increases egg water retention; brooded eggs produce larger, more active, faster swimming and faster developing neonates (*Python regius*, Aubret et al. 2005).
(c) Offspring waste removal (e.g., feces eating)
(d) Increased egg oxygenation (e.g., fanning, brood pumping)
(e) Offspring physiochemical adjustment
(f) Increased offspring immune function
• Paternal presence at the nest increases offspring immune response and likelihood of breeding during the following year in great tits (Tinne et al. 2005)
• Increased maternal provisioning in the Gouldian finch increases offspring immune function (*Erythrura gouldiae*, Pryke and Griffith 2010)
(g) Protection from parasites, parasitoids and disease
• Sand martins that are more heavily infested with ticks have shorter wing length (*Riparia riparia*, Szép and Møller 1999).
(h) Reduction of offspring energetic expenditure (e.g., carrying, thermoregulation)
• Incubation by both parents (vs. incubation by only the mother) results in larger, more developed young, which is thought to affect subsequent survival in the cichlid *Eretmodus cyanostictus* (Grueter and Taborsky 2004).
• Striped mice fathers provide care by huddling with their young in some populations; male care in these populations increases early growth of offspring, which is thought to have effects throughout development and adulthood (*Rhabdomys pumilio*, Schradin and Pillay 2005).
(i) Behavioral support of offspring during intra-specific interactions
• Male baboons support their juvenile offspring during interactions with conspecifics; this support likely contributes to rank acquisition and protects juveniles from injury (Buchan et al. 2003) and is thus likely to affect subsequent reproductive success of offspring.
• In Siberian jays, the presence of fathers in the territory reduces the competitive interference experienced by offspring, which facilitates delayed dispersal and potentially improves offspring fitness (Ekman and Griesser 2002).
(j) Teaching or facilitation of learning
• Golden lion tamarins provision weaned young; in additional to direct nutritional benefits, young also gain informational benefits regarding appropriate food types and handling techniques. Such knowledge improves foraging, survival, and quality throughout life (*Leontopithecus rosalia*, Rapaport 2006).
(k) Inheritance of resources
• Offspring of the spider *Amaurobious ferox* inherit their mother's web after her death; the clutch's collective prey capture is more effective when young are allowed to stay on the maternal web in comparison with cases in which offspring had to construct their own web (Kim 2005).
**(3) Parents ↑ offspring reproductive success during the stage in which parents and offspring are associated**
(a) Provisioning (e.g., lactation, preparation of food, feeding of captured prey)
(b) Protection from parasites, parasitoids, and disease
(c) Behavioral support of offspring during intraspecific interactions
• Vervet monkey females who remain associated with their mothers have higher reproductive success than those females who do not (Fairbanks and McGuire 1986).
• Dominance rank in spotted hyena offspring is positively correlated with maternal dominance rank and this relationship appears to be the result of behavioral support that mothers provide their offspring while acquiring and maintaining dominance status. Dominance rank in turn affects the reproductive success of those offspring (*Crocuta crocuta*, Hofer and East 2003; East et al. 2009).
**(4) Parents manipulate offspring development rate, which increases overall offspring survival or reproductive success across multiple life-history stages**
(a) Parents ↓ the relative amount of time offspring spend in relatively dangerous stages and ↑ the *relative amount of time spent in relatively safe stages*
• Females of the egg-carrying spitting spider *Scytodes pallida* adjust hatching time of eggs in response to the threat of predation (Li 2002).
(b) Parents increase offspring maturation rate
• Parental care in burying beetles, *Nicrophorus vespilloides*, decreases the duration of the larval stage (Smiseth et al. 2003; Lock et al. 2004).
• Paternal yellow baboons repeatedly support their immature offspring during antagonistic interactions; the presence of the father in the offspring's social group accelerates the timing of offspring's physical maturation. Earlier maturation is expected to increase the offspring's lifetime reproductive success (*Papio cynocephalus*, Charpentier et al. 2008).
• Increased maternal provisioning in the Gouldian finch results in offspring fledging earlier (*Erythrura gouldiae*, Pryke and Griffith 2010).

Parental care can improve offspring performance in several ways. First, parents can increase offspring survival during the stage in which they are associated with offspring. This is a particularly well-documented benefit of parental care that can arise through a variety of mechanisms (reviewed in Clutton-Brock 1991; Alonso-Alvarez and Velando 2012; and Table 1). For example, guarding, provisioning, and protection from parasites during particular life-history stages increases offspring survival in numerous species (Table 1). Second, parental care can improve some aspect of offspring quality, which in turn leads to a subsequent increase in offspring survival and/or reproductive success when parents and offspring are no longer associated in close proximity (reviewed in Clutton-Brock 1991 and Alonso-Alvarez and Velando 2012). Such carryover effects have also been well documented in a number of animals, although they are often ignored in parental investment theory for simplicity (Clutton-Brock 1991; Alonso-Alvarez and Velando 2012; Table 1). In great tits, for example, paternal presence at the nest increases offspring immune function and the likelihood of offspring breeding during the following year (Tinne et al. 2005). Likewise, parents can in some cases alter offspring phenotype to cope with particular environmental conditions that offspring are likely to experience in the future (reviewed in Alonso-Alvarez and Velando 2012). For example, in sticklebacks, maternal exposure to a predator during egg laying leads to increased antipredator behavior in offspring posthatching (Giesing et al. 2011). Third, if parent(s) and offspring remain in close contact into adulthood, parents can directly increase their offspring's reproductive success (and hence their inclusive fitness) during the stage in which parents and offspring are associated and in close proximity by either aiding their offspring in mating, reproduction, or by providing resources to grandchildren. Extended family living is relatively rare, and thus, this benefit is likely to be less common than the previous two types of benefits (but see Lee 2003 and Johnstone and Cant 2010). However, mothers have been found to increase their offspring's reproductive success in some mammals (Fairbanks and McGuire 1986; Hofer and East 2003; East et al. 2009). For example, female vervet monkeys are subjected to less aggression, have a greater pregnancy rate, and higher production of surviving infants if their mothers are present in the same troop versus the case in which their mothers are absent (Fairbanks and McGuire 1986).

In general, these three types of benefits have been relatively well studied, and numerous mechanisms have been found to give rise to such benefits (reviewed in Table 1). A fourth benefit, which has received less attention (Table 1), involves parental control over offspring development rate across multiple life-history stages. Offspring developmental rate is a key component of overall offspring performance, and as such, it is likely that parents can improve offspring fitness by manipulating offspring development. Specifically, parents potentially increase overall offspring survival by adjusting the relative amount of time offspring spend in various life-history stages in response to expected offspring mortality. Such a benefit could act alone or in combination with the other general benefits described previously. We outline this hypothesis below.

Natural selection is expected to favor spending relatively little time in life-history stages that are associated with high mortality (Williams 1966; Shine 1978, 1989; Werner and Gilliam 1984; Werner 1986; Remeš and Martin 2002; Warkentin 2007). Empirical evidence supports this prediction. For example, numerous studies have demonstrated plasticity in hatching time in amphibians, reptiles, fishes, and invertebrates in response to perceived mortality risk (e.g., predation or pathogens; reviewed in Warkentin 2007). Similarly, a comparative study of passerine birds found a positive relationship between predation rates and growth rates, and species with higher predation rates fledged at a lighter mass (Remeš and Martin 2002). Such plasticity in hatching time suggests that embryos of many animals can assess current environmental conditions via chemical or physical cues (e.g., mechanical stimulation by predators; Warkentin 2007). There are, however, constraints on how well embryos can assess environmental conditions and/or alter development rate (discussed in Warkentin 2007). In some animals, it is likely that parents are better able to assess current environmental conditions than embryos and may have direct control over offspring developmental rate (see also Wells 2007). Such control would potentially allow parents to retain offspring in relatively safe stages if the environment is currently unfavorable for later developmental stages, or speed up the development of offspring if the environment is favorable for later stages. Such effects on developmental rate could be associated with other benefits of care (e.g., those associated with increased provisioning or guarding; see e.g., Lock et al. 2004). Alternatively, in some cases, parental control of offspring developmental rate might represent the sole benefit of parental care.

The four general benefits of care that we have summarized are similar in that they all potentially increase the lifetime reproductive success of offspring. However, the benefits differ with respect to (1) which life-history stage they influence (e.g., the stage in which parents and offspring are associated or subsequent stages) and (2) the specific effect that they have on offspring (i.e., increased survival, quality, or altered developmental rate). As mentioned earlier, a given form of care might result in multiple benefits. However, stage-specific life-history conditions (e.g., survival, maturation, and reproductive rates) affect the fitness associated with care (Klug and Bonsall 2010). Thus, it is thus important to consider how the benefits of care that occur in different or multiple life-history stages influence the evolution of parental care. In particular, we argue that it is critical to understand how parental manipulation of offspring developmental rate can influence the origin of care, as this benefit is rarely considered in empirical and theoretical studies (see Table 2).

**Table 2:** Life-history trade-offs associated with parental care (*c*) and initial investment in eggs (1*-d*_*Eo*_ and 1*-d*_*Emo*_). Initial investment in eggs is assumed to be costly; as initial investment in eggs increases, parental death increases and parental reproductive rate decreases. Providing parental care is also costly, such that as care increases, parental death rate increases and parental reproductive rate decreases. We consider 11 parental care strategies that are associated with various benefits to offspring.

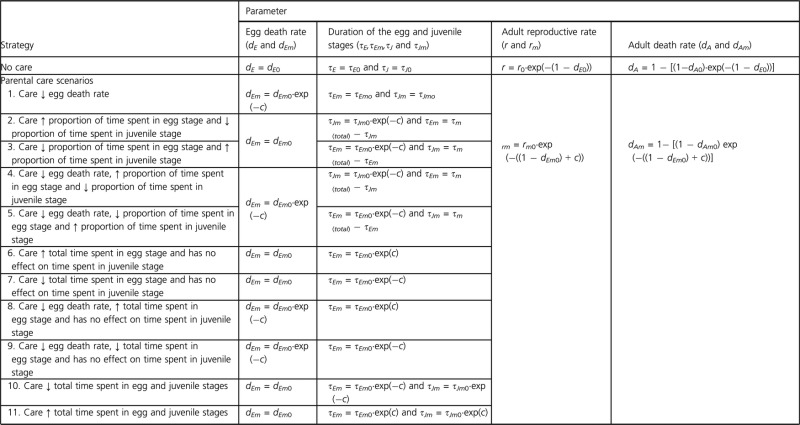

Despite being poorly studied, there is some evidence suggesting that parents can control offspring developmental rate. In burying beetles, *Nicrophorus vespilloides*, parental provisioning is associated with a decrease in the duration of the larval stage (i.e., faster larval development), and there is strong selection for faster larval development (Lock et al. 2004). In this species, removal of the parental female during the first 48 h of development significantly reduces larval growth (Smiseth et al. 2003). Likewise, in the egg-carrying spider *Scytodes pallida* mothers adjust hatching time of eggs in response to the threat of predation (Li 2002). Both females and offspring potentially benefit from earlier hatching of eggs due to the decreased risk of predation. However, it is unclear if this translates into a net benefit for offspring, as there are also potential costs of hatching at a smaller size (Li 2002). Likewise, paternal presence in the social group of immature yellow baboons increases offspring maturation rate (Charpentier et al. 2008), and increased maternal provisioning in the Gouldian finch results in offspring fledging earlier (Pryke and Griffith 2010). In many species, offspring of malnourished mothers who are provisioned less have reduced growth rates (Wells 2007 and references therein). Such early growth restriction is often viewed as a parental and/or offspring adaptation to poor environments (thrifty phenotype hypothesis: reviewed in Wells 2003, 2007). Regardless, these findings suggest that there is typically plasticity in offspring developmental rate, and it is likely, at least in part, that this is under parental control in many species (Smiseth et al. 2003; Lock et al. 2004).

The hypothesis that parental control of offspring development can represent a major benefit of care is a more general form of the ‘safe-harbor’ hypothesis proposed by Shine (1978). Shine (1978) noted that there is a positive correlation between propagule size and the presence of parental care in animals. As an explanation for this pattern, Shine (1978, 1989) suggested that parents can (1) make the egg stage relatively safe for offspring by providing parental care, (2) increase the amount of time offspring spend in the egg stage by producing large eggs, and in doing so, (3) decrease the proportion of time offspring spend in the relatively “high risk” juvenile stage. In contrast to Shine's hypothesis, we do not necessarily assume that parental care is what makes the egg stage relatively safe. Parental care is one factor that might reduce mortality of eggs; however, the egg stage will sometimes be associated with relatively high survival simply because of environmental and ecological factors (e.g., reduced competition or predation, more favorable environmental conditions). Likewise, under some conditions, subsequent stages of development will be associated with relatively high survival. Also in contrast to Shine (1978), we argue that parental manipulation of offspring developmental rate can be the primary benefit of parental care in some cases. We suggest that parents can potentially alter offspring developmental rate through a range of mechanisms, including increased egg provisioning (as suggested by Shine 1978; see also Wells 2007), and through various chemical or behavioral cues or mechanisms during the egg stage (e.g., increased waste removal, oxygenation, or physical stimulation of young).

Manipulation of offspring developmental rate is rarely included in models of the evolution of care and it is thus unclear if it can potentially represent a major, or even the, sole benefit of parental care. As such, it is unknown whether accounting for this benefit can alter our understanding of the evolution of care. In this study, we use a mathematical model to evaluate whether parental control over offspring developmental rate increases offspring and parental fitness. Specifically, we consider the scenarios in which parental care of eggs serves to (1) increase egg survival, (2) increase development rate during a life-history stage with relatively high mortality, and (3) both increase egg survival and increase development rate during a life-history stage with relatively high mortality. As mentioned earlier, parental care is likely associated with multiple benefits in nature. However, considering each benefit in isolation allows us to explore the life-history conditions under which each type of benefit will favor parental care. For each case, we explore the life-history conditions (stage-specific mortality rates) under which the form of care will be favored. This, in turn, allows us to address two broad questions: (1) does the general benefit of care received influence the conditions under which care originates, and (2) if so, can considering beneficial parental manipulation of offspring development rate alter our more general understanding of the evolution of parental care? In answering these questions, our theoretical analyses provide a set of novel and testable predictions regarding when the evolution of parental care is most likely to be favored in relation to benefits of parental care.

## Methods

Using an evolutionary ecology modeling approach (Metz et al. 1992, 1996; Dieckmann and Law 1996; Vincent and Brown 2005; Otto and Day 2007), we allow a rare mutant that exhibits parental care of eggs to invade a resident population in which parental care is absent. While we focus on parental care of eggs, our general approach and findings are applicable to any system in which parental care is provided during some early life-history stage. We assume that the resident strategy is in equilibrium and the alternative parental care strategy attempts to invade from rare into the population. Specifically, we model a stage-structured system in which individuals pass through egg and juvenile stages (although, again, this framework is applicable to any organisms that pass through multiple early life-history stages) and then mature and reproduce as adults. The modeling framework described herein is an extension of our previous work (Klug and Bonsall 2007, 2010; Bonsall & Klug 2011a,b2011b; Klug et al. 2013), and using this framework is ideal in that it allows us to compare the predictions of this model to our previous findings on this topic. This framework also allows us to account explicitly for dynamics associated with various life-history stages.

We focus only on parental care of eggs in this study. We assume that residents and mutants who provide parental care experience the same baseline conditions (i.e., the same death, maturation, and reproductive rates when no care is provided). Parental care is then assumed to be associated with (1) potential benefits to offspring (i.e., either increased survival beyond the baseline survival rate in the absence of care and/or altered developmental rate during the egg and juvenile stages) and (2) costs to the parent providing it (i.e., decreased parental survival and future reproduction relative to the no care scenario; costs and benefits of care described in detail below). For a series of benefits and costs of parental care (described below), we explore the conditions under which parental care is most likely to be able to invade a resident strategy of no care.

### Model dynamics

Individuals pass through an egg (*E*), juvenile, and adult stage (*A*). Eggs increase as adults reproduce and decrease as eggs mature or as eggs die, such that


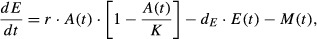
(1)

where *r* represents the rate of egg fertilization (i.e., mean reproductive rate of adults), *d*_*E*_ represents death rate of eggs, and *M*(*t*) is the stage-specific maturation term. We assume logistic population growth, where *K* represents population carrying capacity, and density dependence associated with resource competition affects adult reproduction. Eggs mature after they survive and pass through the stage, such that


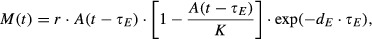
(2)

where *τ*_*E*_ is a time delay representing the duration of the egg stage. Adults in the population increase as eggs mature and pass through the juvenile stage, and decrease as adults die, such that


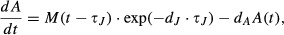
(3)

where *τ*_*J*_ is a time delay representing the length of the juvenile stage, *d*_*J*_ is juvenile death rate, and *d*_*A*_ is the density-independent death rate of adults. Total development time of the resident strategy is assumed to have some fixed duration (i.e., *τ*_*E*_ + *τ*_*J*_ = *τ*_total_).

At equilibrium, the densities of the resident are


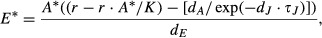
(4)

and


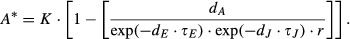
(5)

### Costs and benefits of parental care and initial allocation into eggs

Parents can affect offspring survival and quality by (1) investing energy and nutrients into eggs (which we refer to as initial egg allocation) and (2) providing postfertilization or postoviposition parental care behavior to offspring in a given life-history stage (which we refer to as parental care). Both initial egg allocation and parental care can be associated with benefits to offspring and costs to parents. In the model, such costs and benefits are represented mathematically through the incorporation of trade-off constraints on the resident and mutant dynamics (described below and in Table 2; see also Klug and Bonsall 2010) and as noted, we focus only on parental care associated with the egg stage in this manuscript.

Egg death rate in the absence of care is used as our proxy of initial egg allocation. Thus, by definition, egg death rate is assumed to decrease as initial investment in eggs increases. Initial egg allocation is expected to be costly to both resident and mutant parents, such that as initial egg allocation increases, adult death rate increases and reproductive rates decrease (Table 2). Likewise, providing parental care is assumed to be costly to parents. The level of parental care is approximated by a fixed value, *c* (Table 2), and can be thought of as some average level of care that a mutant adult provides to its own offspring. Providing care is assumed to be costly to parents who exhibit care (i.e., adult mutants), and as the level of care increases, adult survival declines (i.e., death rate increases), and reproductive rate decreases (Table 2).

We consider two general benefits of parental care: (1) parental care of eggs decreases egg death rate (i.e., mutant egg death rate decreases as *c* increases; Table 2) and/or (2) parental care increases or decreases the proportion or absolute amount of time spent in the egg stage (i.e., the proportion or absolute amount of time mutant offspring spend in the egg stage increases or decreases as *c* increases; Table 2). As mentioned earlier, while we focus on the egg stage, this approach is consistent with parental care that influences any early life-history stage. There are eleven possible parental care scenarios (Tables 2 and 3). The first six scenarios focus on the case in which overall maturation time is fixed. When maturation time is fixed (*τ*_*E*_ + *τ*_*J*_ = *τ*_total_), increasing or decreasing time spent in the egg stage will have the opposite effect on the juvenile stage, such that total maturation time is unchanged. For this case, we explored the following scenarios in which care serves to increase offspring egg survival and/or alter relative developmental rates: parental care (1) decreases egg death rate, (2) increases the proportion of time spent in the egg stage and decreases the proportion of time spent in the juvenile stage, (3) decreases the proportion of time spent in the egg stage and increases the proportion of time spent in the juvenile stage, (4) decreases egg death rate, increases the proportion of time spent in the egg stage and decreases the proportion of time spent in the juvenile stage, and (5) decreases egg death rate, decreases the proportion of time spent in the egg stage, and increases the proportion of time spent in the juvenile stage. It is also plausible that maturation time is not fixed. As such increasing or decreasing the duration of the egg stage will have no effect on the duration of the juvenile stage. For this case, we consider four scenarios: parental care (6) increases the time spent in the egg stage but has no effect on time spent in the juvenile stage, (7) decreases the time spent in the egg stage but has no effect on time spent in the juvenile stage, (8) decreases egg death rate and increases the time spent in the egg stage (but has no effect on time spent in the juvenile stage), and (9) decreases egg death rate and decreases the time spent in the egg stage (but has no effect on time spent in the juvenile stage). It is also possible that altering development rate in the egg stage is positively related to development rate in the juvenile stage, and as such we also consider two additional scenarios: parental care (10) decreases the total time spent in the egg and juvenile stages, and (11) increases total time spent in the egg and juvenile stages.

**Table 3:** The life-history conditions (egg and juvenile mortality in the absence of any parental) care that favor the evolution of eleven parental care scenarios

	Conditions under which parental care is most strongly favored	
	
Function of parental care	Egg death rate	Juvenile death rate
(1) Care ↓ egg death rate	High	No effect of juvenile death rate
(2) Care ↑ proportion of time spent in egg stage & ↓ proportion of time spent in juvenile stage	Low	High
(3) Care ↓ proportion of time spent in egg stage & ↑ proportion of time spent in juvenile stage	High	Low
(4) Care ↓ egg death rate, ↑ proportion of time spent in egg stage, & ↓ proportion of time spent in juvenile stage	High & Low	High
(5) Care ↓ egg death rate, ↓ proportion of time spent in egg stage, & ↑ proportion of time spent in juvenile stage	High	Low
(6) Care ↑ total time spent in egg stage & has no effect on time spent in juvenile stage	Care not favored	Care not favored
(7) Care ↓ total time spent in egg stage & has no effect on time spent in juvenile stage	High	No effect of juvenile death rate
(8) Care ↓ egg death rate, ↑ total time spent in egg stage & has no effect on time spent in juvenile stage	Care not favored	Care not favored
(9) Care ↓ egg death rate, ↓ total time spent in egg stage & has no effect on time spent in juvenile stage	High	No effect of juvenile death rate
(10) Care ↓ total time spent in egg and juvenile stages	High & Low	High
(11) Care ↑ total time spent in egg and juvenile stages	Care not favored	Care not favored

The trade-offs associated with each scenario are outlined in Table 2. All of the trade-offs in Table 2 are nonlinear, as nonlinear trade-offs are often more biologically realistic (Clutton-Brock 1991; Alonso-Alvarez and Velando 2012). However, we also consider linear trade-offs and present those results in the Appendix A. Regardless, it is important to note for the case in which parents are able to manipulate offspring development rate, we implicitly assume that the offspring allow the parent to manipulate their development (i.e., there is no parent–offspring conflict over development). Additionally, in order to isolate the direct effects of parental manipulation of offspring development rate on the fitness associated with parental care, we assume no additional costs or benefits of egg or juvenile developmental rate to parents or offspring (e.g., we assume no trade-off between development rate and future offspring survival; see also Discussion).

The trade-off functions described in Table 2 provide some insight into the costs and benefits associated with care; however, these trade-off functions alone do not provide information on whether parental care will be able to invade a resident strategy of no care given the stage-structured life-history conditions and the ecological dynamics of the system. Details on the evolution of parental care from an ancestral state of no care necessitate further analyses and is described below and in Appendix B.

### Invasion dynamics and fitness

By incorporating the relevant trade-offs into the mutant and resident populations (Table 2), the dynamics of the rare mutant are given by:


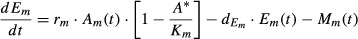
(6)


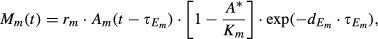
(7)


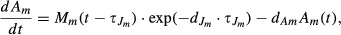
(8)

where A* is the equilibrial abundance of the resident adult population. The other parameters are as described previously, and subscript _*m*_ denotes the new mutant strategy that exhibits parental care. As mentioned previously, to consider the invasion of parental care from an ancestral state of no care, we consider the case in which a rare adult mutant is present and able to provide parental care to its offspring. Thus, we assume that mutant parents are associated with their offspring (e.g., due to spatial clumping or kin recognition) and remain alive long enough to provide care to young. Parental care is only provided by the mutant parent to the mutant offspring (Table 2). For simplicity, the model is thus consistent with haploid inheritance. In contrast, competition for resources that limit reproduction (e.g., food, mating opportunities) occurs more globally; the mutant is assumed to be rare in the population, and thus, density dependence operating on adult mutant reproduction occurs through competition with the resident (eqns. 6, 7).

To explore the benefits that are likely to allow the invasion of parental care, we calculate the fitness of the mutant strategy relative to that of the resident strategy for each of the eleven scenarios described above and in Table 2 using standard invasion analysis (see Appendix B for further details).

## Results

### Fitness of parental care under weak selection

A limiting case for the model can be derived when selection is assumed to be weak (i.e., when the difference between the resident and mutant fitness is small). This scenario provides insight into which life-history traits are most likely to influence the invasion of parental care. When selection is weak, fitness of the mutant strategy is positive when:


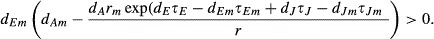
(9)

Fitness is expected to increase with increasing reproductive rate (*r*_*m*_) and decline as the reproductive rate of the resident increases (*r*). In the context of the hypotheses considered here, alterations in mutant strategy egg (*τ*_*Em*_) and juvenile development (*τ*_*Jm*_), as well as egg death rate (*d*_*Em*_) are expected to affect fitness. In particular, it is expected that in the demographic limit (i.e. when there are no costs of developing quickly and no trade-offs between time spent in the egg and juvenile stages), the egg and juvenile development times should evolve to be as short as possible to minimize costs associated with being in different stage or age classes and maximize reproductive potential (as 
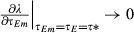
). Under weak selection, differences between mutant (*K*_*m*_) and resident (*K*) carrying capacities have little influence on mutant fitness and hence have little effect on the evolution of parental care.

### Parental manipulation of offspring development can be a major benefit of parental care

We next examined fitness associated with parental care for each of the eleven general care scenarios above (see also Tables 2 and 3). Fitness can be either positive (i.e., the fitness of the mutant is greater than that of the resident), negative (i.e., the fitness of the mutant strategy is less than that of the resident), or zero (i.e., the fitness of the mutant and resident are equal). We would expect parental care to evolve when fitness is positive.

#### Parental care alters relative time spent in egg and juvenile stages

**Figure 1:**
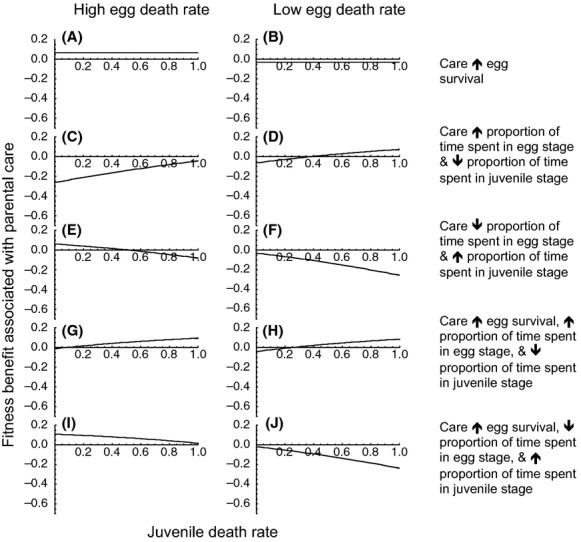
Parental care that only increases egg survival will be favored when egg death rate in the absence of care is high (A) but not when egg death rate in the absence of care is low (B) regardless of juvenile death rate. Parental care that increases the proportion of time spent in the egg stage and reduces time spent in the juvenile stage results in fitness losses when egg death rate is high (C) but will be favored when egg death rate is low and juvenile death rate is high (D). Parental care that decreases the proportion of time spent in the egg stage and increases the proportion of time spent in the juvenile stage will be favored when egg death rate is high (E) but not when it is low (F). Parental care that increases egg survival, increases the proportion of time spent in the egg stage, and decreases the proportion of time spent in the juvenile stage will be favored at both high and low egg death rates and across a broad range of juvenile death rates (G, H). Parental care that increases egg survival, decreases the time spent in the egg stage, and increases time spent in the juvenile stage will be favored when egg death rate in the absence of care is high (I) but not when it is low (J). Unless otherwise noted, *r*_0_ = *r*_*m*0_, *d*_*A*0_ = *d*_*Am*0_ = 0.5, *K* = *K*_*m*_, *τ*_*E*0_ = *τ*_*Em*0_ = 5, *τ*_*J*0_ = *τ*_*Jm*0_ = 5, *c* = 0.4, *d*_*J*0_ = *d*_*Jm*0_, *d*_*E*0_ = *d*_*Em*0_. High egg death rate: *d*_*Em*0_ = 0.9; low egg death rate: *d*_*Em*0_ = 0.1.

**Figure 2:**
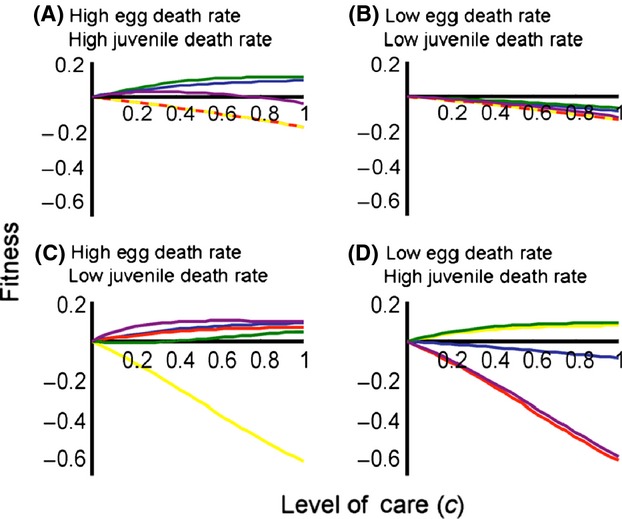
Fitness benefits of various levels of parental care (*c*) when A) baseline egg and juvenile mortality are high (*d*_*Em*0_ = *d*_*Jm*0_ = 0.9), B) baseline egg and juvenile mortality (*d*_*Em*0_ and *d*_*Jm*0_) are low (*d*_*Em*0_ = *d*_*Jm*0_ = 0.1), C) baseline egg mortality is high and juvenile mortality is low (*d*_*Em*0_ = 0.9, *d*_*Jm*0_ = 0.1), and D) baseline egg mortality is low and juvenile mortality is high (*d*_*Em*0_ = 0.1, *d*_*Jm*0_ = 0.9). We consider the following scenarios: (1) parental care decreases egg death rate (blue line), (2) parental care increases the proportion of time spent in the egg stage (yellow line), (3) parental care increases the proportion of time spent in the juvenile stage (red line), (4) parental care decreases egg death rate and increases the proportion of time spent in the egg stage (green line), and (5) parental care decreases egg death rate and increases the proportion of time spent in the juvenile stage (purple line). Unless otherwise noted, *r*_0_ = *r*_*m*0_, *d*_*A*0_ = *d*_*Am*0_ = 0.5, *K* = *K*_*m*_, *τ*_*E*0_ = *τ*_*Em*0_ = 5, *τ*_*J*0_ = *τ*_*Jm*0_ = 5, *c* = 0.4, *d*_*J*0_ = *d*_*Jm*0_, *d*_*E*0_ = *d*_*Em*0_.

If parental care decreases the death rate of eggs (Scenario 1), parental care is favored when egg death rate in the absence of care is high (Fig. 1A). Indeed, when egg death rate in the absence of care is high, any level of parental care is expected to evolve (Fig. 2A and C, blue lines). In contrast, parental care is never expected to evolve if egg survival in the absence of care is high (Figs 1B and 2 B,D, blue lines).

If parental care increases the relative amount of time spent in the egg stage and decreases the proportion of time spent in the juvenile stage (but has no effect on egg death rate and does not alter total development time; Scenario 2), all levels of parental care will be favored when egg death rate in the absence of care is relatively low and juvenile death rate is relatively high (Fig. 1D and yellow line in Fig. 2D). In contrast, care that increases relative time spent in the egg stage and decreases relative time spent in the juvenile stage will not evolve when egg and juvenile mortality are equivalent (Fig. 2A and B, yellow line) or when egg mortality is relatively high (Fig. 1C and yellow line of Fig. 2C).

Parental care that increases the proportion of time spent in the juvenile stage and decreases the proportion of time in the egg stage (but has no effect on egg death rate and does not alter total offspring development time; Scenario 3) will evolve when juvenile mortality is relatively low and egg mortality is relatively high ( Fig. 1E). Under such conditions, all levels of parental care can result in fitness gains (Fig. 2C, red line). Parental care that increases the relative duration of the juvenile stage and decreases the relative duration of the egg stage will never occur if juvenile mortality is high (Fig. 1E and F and red lines of Fig. 4A and D) or when egg and juvenile morality are equal (Fig. 2A and B, red lines).

If parental care decreases offspring mortality, increases the proportion of time spent in the egg stage, and decreases the proportion of time spent in the juvenile stage (Scenario 4), parental care will be most strongly favored when juvenile death rate is high, regardless of baseline egg death rate (Fig. 1G and H and green lines of Fig. 2A and D). However, when juvenile death rate is low and eggs cannot survive well without care, high levels of parental care may be selected for (Fig. 2C, green lines). Parental care that increases offspring survival, increases the relative duration of the egg stage, and decreases the relative duration of the juvenile stage will never evolve if both egg and juvenile death rates are relatively low (Fig. 1G and H).

Parental care that decreases offspring mortality, increases the proportion of time spent in the juvenile stage, and decreases the proportion of time spent in the egg stage (Scenario 5) will be expected to evolve when egg death rate is high (Fig. 1I), particularly when juvenile mortality is low (Fig. 2C, purple line). Parental care that increases offspring survival and the relative duration of the juvenile stage will never occur if egg death rate is low, regardless of the level of juvenile mortality (Fig. 1J and purple lines of Fig. 2B and D).

#### Parental care alters absolute amount of time spent in egg stage

**Figure 3:**
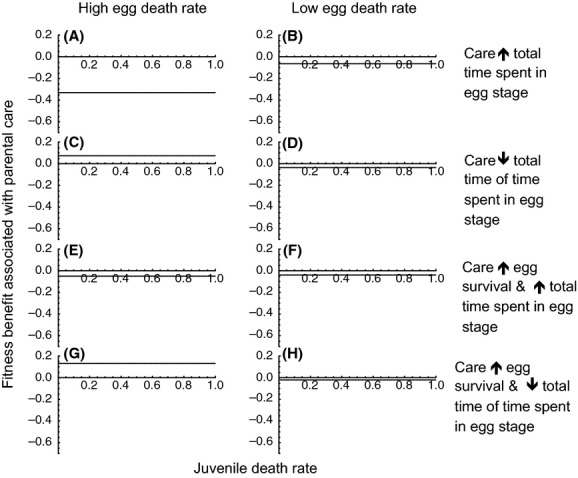
Parental care that increases that increases the time spent in the egg stage but has no effect on time spent in the juvenile stage will not be favored at high (A) or low (B) egg death rates. Parental care that decreases the time spent in the egg stage but has no effect on juvenile time will be favored when egg death rate is high (C) but not when it is low (D). Care that both increases egg survival and increases time spent in the egg stage (but has no effect on juvenile time) will not be favored at high (E) or low (F) egg death rates. Care that increases egg survival and decreases time spent in the egg stage (but has no effect on juvenile time) will be favored when egg death rate is high (G) but not when it is low (H). Unless otherwise noted, *r*_0_ = *r*_*m*0_, *d*_*A*0_ = *d*_*Am*0_ = 0.5, *K* = *K*_*m*_, *τ*_*E*0_ = *τ*_*Em*0_ = 5, *τ*_*J*0_ = *τ*_*Jm*0_ = 5, *c* = 0.4, *d*_*J*0_ = *d*_*Jm*0_, *d*_*E*0_ = *d*_*Em*0_. High egg death rate: *d*_*Em*0_ = 0.9; low egg death rate: *d*_*Em*0_ = 0.1

We next consider the case in which parental care serves to alter egg developmental rate but has no effect on developmental rate of juveniles (Fig. 3A–H). Parental care that decreases the amount of time spent in the egg stage (thereby decreasing overall development time) but has no influence on time spent in the juvenile stage (Scenario 7) will be favored if egg mortality is high (Fig. 3C) but not when egg mortality is low (Fig. 3D). Likewise, parental care will be favored if there are multiple functions of care, such that care increases egg survival and decreases total time spent in the egg stage (Scenario 9), when egg death rate is high (Fig. 3G) but not when it is low (Fig. 3H). In contrast, parental care that increases the total amount of time spent in the egg stage (Scenario 6) is unlikely to be favored, regardless of whether egg death rate is low or high (Fig. 3A and B). This remains the case when care both increases egg survival and increases time spent in the egg stage (Scenario 8; Fig. 3E and F). In other words, care that increases the overall time spent maturing is unlikely to be favored evolutionarily in the absence of other benefits (e.g., increased quality due to longer development, which are not considered in this modeling framework). In hindsight, this is somewhat unsurprising as there is always some risk of mortality in any developmental stage – as such, the longer an individual spends in a stage, the greater their risk of dying in that stage.

In summary, parental manipulation of offspring development can be a substantial or the only benefit of care even when total development time is not fixed (i.e. when parental manipulation leads to an overall increase or decrease in development time; Fig. 3C and G), although it is less likely to be beneficial in comparison to the cases above in which care alters the proportion of time spent in each stage. In the absence of additional benefits associated with remaining in a stage (e.g., decreased time spent in a relatively dangerous stage, as discussed in the previous section, or increased quality or size which are not considered herein), evolution is unlikely to favor slower development regardless of the danger associated with a given stage.

#### Parental care alters egg and juvenile development in similar ways

**Figure 4:**
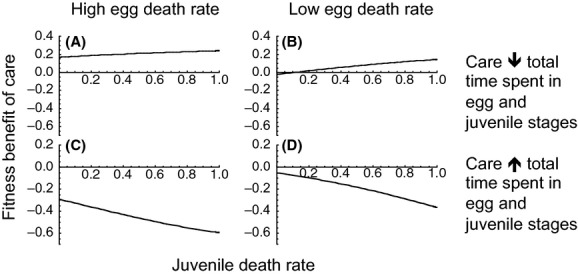
Parental care that decreases the total time spent in both the egg and juvenile stage will be favored at high (A) and low (B) egg death rates. Parental care that increases the total time spent in egg and juvenile stages will not be favored at high (C) or low (D) egg death rates.

In some cases, there might be constraints associated with development such that increasing or decreasing time spent in one stage has a similar effect on the subsequent stage (i.e., speeding up or slowing down egg development speeds up or slows down juvenile development). If decreasing time spent in one stage decreases time spent in the other stage (Scenario 10), parental manipulation of offspring development will be most strongly favored when egg death rate in the absence of care is high (Fig. 4A) and when juvenile mortality is high (Fig. 4A and B). In contrast, slowing down both egg and juvenile development is unlikely to evolve in the absence of additional benefits ( Fig. 4C and D). Again, this occurs as there is always some mortality associated with each life-history stage.

Importantly, our model does not incorporate trade-offs associated with developing quicker. If decreasing development time is associated with costs (e.g., due to smaller size at maturation), we would expect parental manipulation of development to be favored only when the benefits of such manipulation (i.e. the survival benefits associated with spending less time in a risky stage) outweigh the costs associated with increased development rate.

### Parental manipulation of offspring development can change our understanding of parental care

In the previous section, we have shown that parental manipulation of offspring development rate can alone represent a major benefit of parental care under some conditions. Now, the perhaps more interesting question becomes: does accounting for the benefits associated with parental manipulation of offspring development rate change our general understanding of the evolution of parental care?

In short, accounting for parental manipulation of offspring development does alter our understanding of parental care (summarized in Table 3). When the only benefit of parental care is increased offspring survival (Scenario 1), we find that parental care is only expected to be favored when egg death rate in the absence of care is high (Fig. 1A; Table 3), and care is not expected to result in fitness benefits when offspring survive relatively well in the absence of care (Fig. 1B; Table 3). If, however, parental care decreases the proportion of maturation time spent in relatively unsafe stages, parental care can be favored across a broader range of egg death rates (Table 3). Specifically, care that only serves to decrease relative time spent in an unsafe stage will result in fitness gains regardless of how well eggs survive in the absence of care (Fig. 1D and E). If care increases offspring survival and also decreases the proportion of time spent in a dangerous developmental stage, care has the potential to evolve over a relatively broad range of egg death rates (Fig. 1G and I; Table 3). Care also has the potential to evolve across a broad range of egg death rates if increasing developmental rate decreases the time spent in both egg and juvenile stages (Fig. 4A and B; Table 3).

Likewise, when the only benefit of parental care is increased egg survival, juvenile survival does not influence the fitness benefits associated with care (Fig. 1A and B; Table 3). If, however, parents manipulate offspring development to reduce time spent in an unsafe stage and also increase time spent in a safe stage, juvenile survival influences the conditions under which care evolves (Table 3). If juvenile survival is low, parental care that decreases the relative amount time spent in the juvenile stage will be favored (Figs. 1D and 3A and B; Table 3).

In contrast to the cases above in which overall development time is fixed, parental manipulation of offspring development rate that increases overall development rate (and decreases time spent maturing) does not alter the conditions under which care will evolve. Care that decreases time spent in the egg stage but has no effect on juvenile development rate will only be favored when egg death rate is high. This is exactly the condition under which we would expect care that increases egg survival to evolve (Fig. 1A; Table 3). Parental manipulation of offspring development that decreases overall development rate (and increases time spent maturing) is in general unlikely to evolve in absence of additional benefits not considered in this model (Fig. 3A, B, E and F; Table 3).

In summary, accounting for potential benefits associated with parental manipulation of offspring development rate broadens the life-history conditions (i.e., egg and juvenile mortality) under which care can evolve (Table 3). This is particularly the case when total development time is fixed (Scenarios 1-5) and remains true regardless of whether parental manipulation of offspring development rate is the only benefit of care or if parental care is associated with other offspring benefits (i.e., increased egg survival).

These general patterns are robust to different trade-off functions (results associated with linear trade-off functions given in Appendix A).

## Discussion

Here, we have illustrated that (1) parental control over offspring development rate can represent a substantial or even the single benefit of parental care and (2) considering parental manipulation of offspring development rate can broaden the life-history conditions under which we expect care to evolve. The specific finding that parental control of offspring development rate can alone favor the evolution of parental care and/or alter the life-history conditions under which care will be selected for is, to our knowledge, novel and might help explain natural patterns of care.

Parental behavior that decreases time offspring spend in a dangerous stage should be considered a form of parental care (e.g., Figs. 1D and E, 3C), regardless of whether parents engage in additional behavior such as guarding or provisioning (as in Figs. 1H and I and 3G). The general idea that parental control over offspring developmental rate can be adaptive is consistent with previous work. For instance, Shine (1978, 1989) suggested that parents who provide parental care might increase propagule size to increase the duration of time offspring spend in the relatively safe egg stage. Our findings differs from those of Shine (1978, 1989) in that we show that parental manipulation of offspring development that decreases relative time spent in a dangerous stage can be favored even if care is not what makes that stage safe. Also, consistent with our theoretical findings, Lock et al. (2004) found that parental care increases offspring larval development in burying beetles and that this increased developmental rate is selected for (see also Smiseth et al. 2003 and Lock et al. 2007).

Our findings are also consistent with more general life-history theory suggesting that individuals should minimize the time they spend in relatively dangerous life-history stages (Williams 1966; Werner and Gilliam 1984; Werner 1986; Warkentin 2007) and work focused on parental effects. Parents affect the development of their offspring in numerous ways (e.g., in relation to predator behavior, pathogens, and other adverse environmental conditions; reviewed in Alonso-Alvarez and Velando 2012). Previous work has also found that parents can alter offspring development such that they develop more slowly under poor environmental conditions. According to the thrifty phenotype hypothesis and related theory (Wells 2003, 2007), this can be adaptive for offspring if slower development makes them well-suited to a poor environment later in life and/or for parents if it reduces parental investment in an optimal way. Our work regarding parental manipulation of offspring developmental rate differs from the thrifty phenotype hypothesis as we assume that parental manipulation of developmental rate is itself a benefit of care, and we also focus on the case in which parents speed up (rather than slow down) development when the environment is poor.

Whether parental care increases offspring survival (the most well-studied benefit of care; Table 1) or decreases the time spent in a relatively dangerous life-history stage affects the conditions under which parental care will evolve (Table 3), particularly when maturation time is fixed (i.e. when increasing time spent in the egg stage decreases time spent in the juvenile stage, and vice versa). When the sole benefit of parental care is increased offspring survival, parental care is expected to be favored when offspring need care the most—i.e. when offspring survival in the absence of care is relatively low (e.g., Fig. 1A and B; Table 3). This finding is consistent with previous empirical and theoretical work (reviewed in Clutton-Brock 1991; Klug and Bonsall 2010; Royle et al. 2012). In contrast, when care only serves to increase time spent in the egg stage and decreases time spent in the juvenile stage, parental care will be favored when egg mortality is relatively low (e.g., Fig. 1D; Table 3). When care both increases egg survival and increases time spent in the egg stage, care is expected to evolve over a range of egg and juvenile death rates (e.g., Fig. 1G–I; Table 3).

These patterns are not predicted by previous theory, which suggests that care is expected to evolve when egg mortality (or mortality in another early life-history stage) in the absence of care is high (Stearns 1976; Klug and Bonsall 2010). Likewise, the finding that juvenile survival can affect egg-only care is not predicted by previous theory (Klug and Bonsall 2010). If parents decrease egg developmental rate and this affects the time spent in the juvenile stage, juvenile survival will influence whether care is favored. Specifically, if care increases time spent in a relatively safe egg stage and decreases time spent in the juvenile stage, care will be favored when the juvenile stage is associated with high mortality. If, on the other hand, care decreases time spent in a relatively dangerous egg stage and increases time spent in the juvenile stage, care will be favored when the juvenile stage is associated with low mortality.

Importantly, our modeling framework does not assume any costs or benefits of the time spent in a particular stage except those associated with survival. This allows us to determine whether parental manipulation of offspring developmental rate can directly favor the origin of parental care. However, in nature, it is likely that development time is associated with other costs and benefits. Specifically, the cost of egg care will often increase if offspring remain eggs for longer. Similarly, there is very likely a cost to offspring of developing more quickly if fast development is associated with smaller size (reviewed in Warkentin 2007) when compensatory growth is impossible. Such effects of development can be paid over a long time frame (Metcalfe and Monaghan 2001) and set the stage for parent-offspring conflict. Over time, parent/offspring interactions would be expected to co-evolve, and conflict over resource allocation is likely to occur (see, e.g., Royle et al. 2002). For example, if there are costs of rapid development related to smaller size, selection might favor offspring attempting to gain control over resource allocation, particularly if parents control development. Exploring the dynamics between resource allocation, development rate, and parent-offspring conflict would be an interesting avenue of future research. In addition, we have not accounted for carry-over effects of care or within clutch dynamics in our model. Interactions among offspring and between parents and offspring can greatly influence the evolution of parental care (Hinde et al. 2010; Gardner and Smiseth 2011). In the future, it will be key to incorporate such trade-offs in order to determine how the benefits of reducing time spent in a dangerous stage interact with other life-history trade-offs and the costs of care. Regardless, parental manipulation of offspring development that reduces time spent in a dangerous stage is only expected to evolve if the net benefits of such manipulation outweigh any net costs to parents in terms of increased care and any costs to offspring associated with developing quickly.

In summary, parental control over offspring development can independently lead to the origin of parental care when it results in offspring spending less time in relatively dangerous stages. Parental manipulation of offspring developmental rate is a relatively unexplored benefit of care (Table 1) and it will be interesting to examine whether such a benefit exists in future empirical studies. Considering parental manipulation of offspring developmental rate enhances our understanding of how egg and juvenile mortality can influence the origin of care. In general, beneficial parental manipulation of offspring development broadens the life-history conditions under which care can evolve.

## Figures and Tables

**Figure A1:**
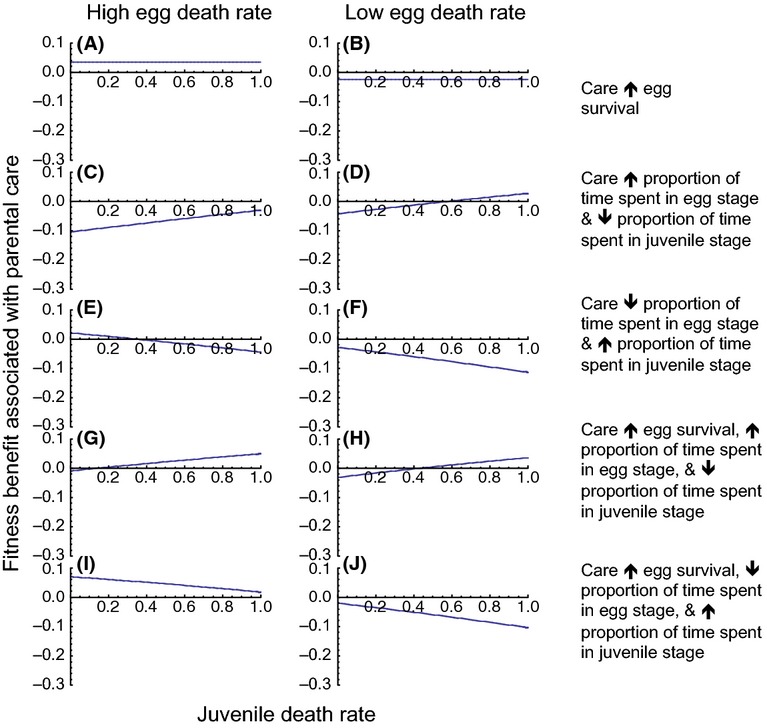
Parental care that only increases egg survival will be favored when egg death rate in the absence of care is high (A) but not when egg death rate in the absence of care is low (B) regardless of juvenile death rate. Parental care that increases the proportion of time spent in the egg stage and reduces time spent in the juvenile stage results in fitness losses when egg death rate is high (C) but will be favored when egg death rate is low and juvenile death rate is high (D). Parental care that decreases the proportion of time spent in the egg stage and increases the proportion of time spent in the juvenile stage will be favored when egg death rate is high (E) but not when it is low (F). Parental care that increases egg survival, increases the proportion of time spent in the egg stage, and decreases the proportion of time spent in the juvenile stage will be favored at both high and low egg death rates and across a broad range of juvenile death rates (G, H). Parental care that increases egg survival, decreases the time spent in the egg stage, and increases time spent in the juvenile stage will be favored when egg death rate in the absence of care is high (I) but not when it is low (J). Unless otherwise noted, *r*_0_ = *r*_*m*0_, *d*_*A*__0_ = *d*_*A*__*m*0_ = 0.5, *K* = *K*_*m*_, *τ*_*E*0_ = *τ*_*Em*0_ = 5, *τ*_*J*0_ = *τ*_*Jm*0_ = 5, *c* = 0.4, *d*_*J*__0_ = *d*_*J*__*m*0_, *d*_*E*__0_ = *d*_*E*__*m*0_. High egg death rate: *d*_*E*__*m*0_ = 0.9; low egg death rate: *d*_*E*__*m*0_ = 0.1.

**Figure A2:**
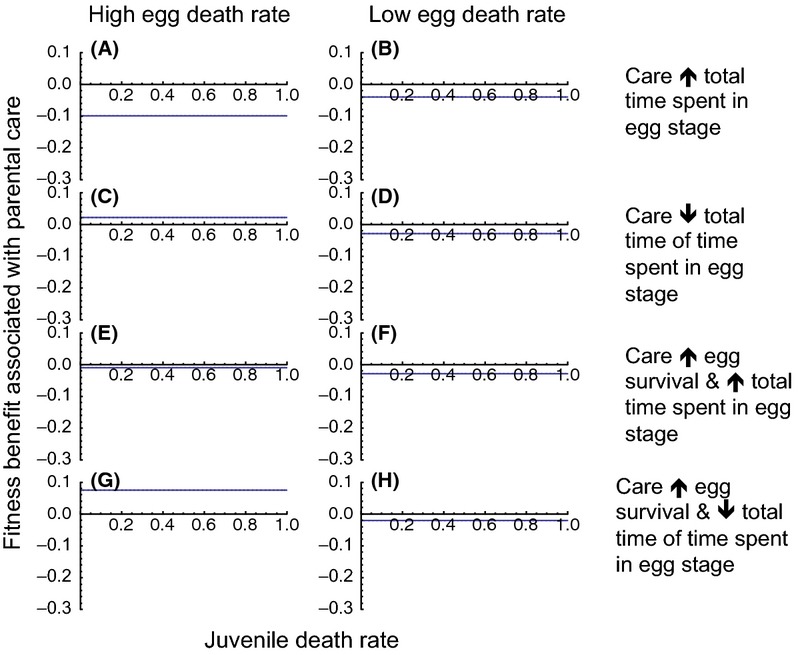
Parental care that increases that increases the time spent in the egg stage but has no effect on time spent in the juvenile stage will not be favored at high (A) or low (B) egg death rates. Parental care that decreases the time spent in the egg stage but has no effect on juvenile time will be favored when egg death rate is high (C) but not when it is low (D). Care that both increases egg survival and increases time spent in the egg stage (but has no effect on juvenile time) will not be favored at high (E) or low (F) egg death rates. Care that increases egg survival and decreases time spent in the egg stage (but has no effect on juvenile time) will be favored when egg death rate is high (G) but not when it is low (H). Unless otherwise noted, *r*_0_ = *r*_*m*0_, *d*_*A*__0_ = *d*_*A*__*m*0_ = 0.5, *K* = *K*_*m*_, *τ*_*E*0_ = *τ*_*Em*0_ = 5, *τ*_*J*0_ = *τ*_*Jm*0_ = 5, *c* = 0.4, *d*_*J*__0_ = *d*_*J*__*m*0_, *d*_*E*__0_ = *d*_*E*__*m*0_. High egg death rate: *d*_*E*__*m*0_ = 0.9; low egg death rate: *d*_*E*__*m*0_ = 0.1

**Figure A3:**
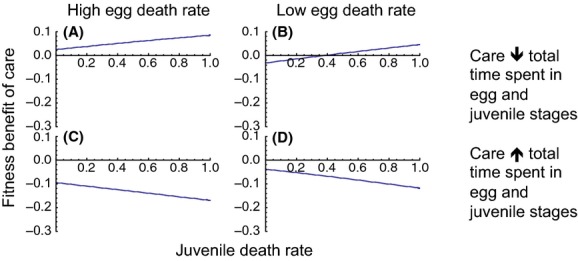
Parental care that decreases the total time spent in both the egg and juvenile stage will be favored at high (A) and low (B) egg death rates. Parental care that increases the total time spent in egg and juvenile stages will not be favored at high (C) or low (D) egg death rates.

## Appendix A: Linear Trade-Off Scenarios

To evaluate whether our patterns are robust to different trade-off functions, we perform analyses for all scenarios (see Methods of main text) using linear trade-offs (Table 4; see main text for non-linear trade-off results). In all cases, our qualitative patterns remain unchanged regardless of whether we consider linear (Figs. 1A–3A) or nonlinear (Fig. 4 in main text) trade-off functions.

**Table A1:** Life-history trade-offs associated with parental care (*c*) and initial investment in eggs (1*-d*_*Eo*_ and 1*-d*_*Emo*_). Initial investment in eggs is assumed to be costly; as initial investment in eggs increases, parental death increases and parental reproductive rate decreases. Providing parental care is also costly, such that as care increases, parental death rate increases and parental reproductive rate decreases. We consider 11 parental care strategies that are associated with various benefits to offspring.

## Appendix B: Invasion dynamics and fitness

By incorporating the relevant trade-offs into the mutant and resident populations (Table 2), the dynamics of the rare mutant are given by:

(S-1)

(S-2)

(S-3)

where *E*_*m*_ and *A*_*m*_ are the dynamics of the egg and adult strategy and *M*_*m*_ is the maturation term from the egg to the adult stage. The subscript _*m*_ denotes the new mutant strategy that exhibits parental care. Other parameters are defined as follows: *r*_*m*_ is the rate of egg fertilization rate, *d*_*Em*_ is the death rate of eggs, *K*_*m*_ is the population carrying capacity, *τ*_*Em*_ is the duration of the egg stage, *τ*_*Jm*_ is the duration of the juvenile stage, *d*_*Jm*_ is the juvenile death rate and *d*_*Am*_ is the density-independent adult death rate. A* is the equilibrial abundance of the resident adult population.

Analysis proceeds by determining when the (fitness) growth rate (λ) of the mutant strategy is positive (λ > 0) and rare (*E*_*m*_*→*0, *A*_*m*_*→*0). As the mutant strategy is rare, any intraspecific density dependence will be weak as *A*_*m*_ *→* 0 so the dynamics (S-1–S-3) can be expressed as

(S-4)

(S-5)

(S-6)

As fitness differences are assumed to be small (and with the approximation exp(−*x*) = 1 – *x*), the lifetime fitness of the mutant (λ) can then be found by taking the determinant of an appropriately formulated Jacobian matrix (**J**):

where the entries in this matrix are the partial derivatives (after taking appropriate Laplace transforms to deal with time lags) associated with the mutant strategy in the presence of the resident strategy (which is at the nontrivial equilibrium, A*).

The determinant is:

By solving this resulting characteristic equation for *λ*, expressions for fitness under weak and strong selection can be derived (eqn. 10).
